# Everything but the squeal: a guide for head and neck surgery training on the live porcine model

**DOI:** 10.1007/s00405-023-07882-5

**Published:** 2023-02-24

**Authors:** Ignacio Alcalá Rueda, Álvaro Sánchez Barrueco, Carlos Cenjor Español, Abel Bogoya Castaño, José Miguel Villacampa Aubá

**Affiliations:** 1 Alfonso X El Sabio University (UAX), Madrid, Spain; 2 Otolaryngology Department, University Hospital General de Villalba, Madrid, Spain; 3 Otolaryngology Department, University Hospital Fundación Jiménez Díaz, Av. de los Reyes Católicos, 2, 28040 Madrid, Spain

**Keywords:** Head and neck, Laboratory animal model, Resident education, Training program, Anatomy, Comparative

## Abstract

**Purpose:**

The porcine model has been demonstrated to be cost-effective for head and neck surgery training. There is no literature describing the porcine head and neck anatomy. The purpose of this study is to provide a porcine surgical guide for training head and neck residents.

**Methods:**

Five head and neck dissections were performed under general anesthesia on the Large White pig model in the animal facilities of the University Hospital Fundación Jiménez. Sessions were photographed, and reference anatomical measurements were taken.

**Results:**

The sternum–chin distance (*x* = 15.80 cm, *σ* = 0.44), chin–chin distance (*x* = 11.10 cm, *σ* = 2.30), prelaryngeal musculature length (*x* = 10.30 cm, *σ* = 1.92) and supraomohyoid triangle area (*x* = 7.07 cm^2^, *σ* = 3.91) were among the measurements obtained. The porcine head and neck anatomy was detailed.

**Conclusions:**

Head and neck porcine anatomy was thoroughly described, with emphasis on the similarities with human anatomy. The porcine model is capable of simulating human anatomy for surgery training.

## Introduction

A challenging learning curve demands specialized training for procedures like tracheotomy, neck dissection and laryngectomy. Head and neck (H&N) surgery is becoming less crucial in some cases due to the introduction of radiation therapy, chemotherapy, and immunotherapy in favor of organ preservation strategies [1]. Due to the greatly reduced number of surgeries that residents in otolaryngology and head and neck surgery can attend, this has a considerable impact on their training. Residents can overcome this problem with the aid of simulation models, which are now receiving more attention from researchers across all surgical specialties, not just in otolaryngology [2–5].

Despite the fact that the porcine model has historically been used as a surgical training tool to improve skills in H&N [6], there is no established approach or implementation methodology for its use in H&N resident training, and it is still being validated.

Amidst the number of publications that discuss how well the porcine model works for various procedures we have not been able to locate a guidebook that explains swine neck anatomy specifically for use in head and neck surgery simulation. Propst et al. [7] detailed manual on handling pigs and instruction in airway surgery was released in 2010; nevertheless, laryngectomy and neck dissection were not included.

We questioned how an otolaryngology resident might start his training in H&N surgery when the majority of operations are difficult cases nowadays (radiated patients, salvage surgery, second tumors, or relapses). In an effort to find a solution, we began developing the live porcine model in 2015 [8]. However, the lack of a trustworthy anatomical guideline presented several challenges.

The goal of this paper is to provide a guide for surgical dissection in the porcine model that allows for the transfer of skills learned in the porcine model to head and neck surgery in humans, with a focus on procedures such as tracheotomy, neck dissection, and total laryngectomy.

## Materials and methods

### Project approval

The IIS-FJD Ethical Committee and the Animal Welfare Committee of the Fundación Jiménez Díaz University Hospital gave their approval to the conducted study. The method for handling animals was inspired by a prior study from our team [8], which discussed and provided details on the progress of the resident skills following training with a live swine model.

### Animal model

Five large white pigs served as models. The animals were between three and six months old and weighed between 20 and 25 kg. A tracheal tube was utilized for ventilation after 5% isoflurane was administered to anesthetize each specimen. An overdose of sodium thiopental was administered to the animal for euthanasia at the conclusion of the surgical simulation session. Adequate measures were taken to minimize pain and discomfort in accordance with the European Communities Council Directive of 24 November 1986 (86/609/EEC) and in accordance with local laws and regulations.

### Data

Table 1 displays the anatomical measurements made while dissecting the pig model. Additionally noted were the location of the tracheostomy ring and the thyroid gland.

**Table 1:** Measurements of the head and neck structures in the porcine model expressed in cm, cm^2^ and cm^3^

Anatomical measure	Mean	Standard deviation
Sternum–chin length (cm)	15.80	0.45
Mental–mento length (cm)	11.10	2.30
Tracheal ring thyroid localization	5º–6º	N/A
Tracheal ring for tracheostomy	3º–4º	N/A
Tracheal diameter (cm)	1.30	0.31
Tracheal length (cm)	3.30	0.45
Platysmal flap length (cm)	8.40	1.14
Strap muscle length (cm)	10.30	1.92
Distance from the upper edge of the omohyoid muscle to the upper edge of the sternomastoid muscle (R) (cm)	3.40	0.96
Distance from the upper edge of the omohyoid muscle to the upper edge of the sternomastoid muscle (L) (cm)	3.54	0.90
SCM length (R) (cm)	10.50	1.80
SCM length (L) (cm)	10.70	1.57
Distance from the upper edge of the sternomastoid muscle until it crossed the omohyoid muscle (R) (cm)	4.90	1.02
Distance from the upper edge of the sternomastoid muscle until it crossed the omohyoid muscle (L) (cm)	4.70	0.97
Omohyoid muscle length (R) (cm)	6.40	0.55
Omohyoid muscle length (L) (cm)	6.00	0.71
Omohyoid muscle length from its intersection with the mastoid muscle to its superior insertion (R) (cm)	4.20	1.04
Omohyoid muscle length from its intersection with the mastoid muscle to its superior insertion (L)	4.10	1.75
Supraomohyoid triangle area (R) (cm^2^)	7.10	3.78
Supraomohyoid triangle area (L) (cm^2^)	7.04	4.07
Submandibular gland height (R) (cm)	1.80	0.27
Submandibular gland height (L) (cm)	1.90	0.42
Submandibular gland length (R) (cm)	4.00	0.71
Submandibular gland length (L) (cm)	4.00	0.71
Submandibular gland width (R) (cm)	1.70	0.57
Submandibular gland width (L) (cm)	1.70	0.57
Submandibular gland volume (R) (cm^3^)	6.01	0.45
Submandibular gland volume (L) (cm^3^)	6.31	0.96
External jugular vein diameter (R) (cm)	0.62	0.11
External jugular vein diameter (L) (cm)	0.56	0.13
Internal jugular vein diameter (R) (cm)	0.32	0.04
Internal jugular vein diameter (L) (cm)	0.34	0.05
Common carotid artery diameter (R) (cm)	0.38	0.13
Common carotid artery diameter (L) (cm)	0.38	0.13
Epiglottis height (cm)	3.70	0.45
Epiglottis width (cm)	3.90	0.42
Larynx height (cm)	9.00	0.35
Larynx width (anteroposterior diameter) (cm)	3.30	0.45
Larynx width (lateral diameter) (cm)	3.14	0.50

For some of the measurements, right (R) and left (I) sides are specified

The area of the supraomohyoid triangle, which matched that of a functional neck dissection in a human, was calculated using these measurements and the Heron formula. The distance from the upper edge of the omohyoid muscle to the upper edge of the sternomastoid muscle was taken to measure side (a). The distance from the upper edge of the sternomastoid muscle till it crossed the omohyoid muscle was employed to measure side (b). The length of the omohyoid muscle from its intersection with the mastoid muscle to its superior insertion was determined for side (c) (Figs. 2,3):

$$\left(\text{Heron Formula}\right)\mathrm{\acute{A} }=\sqrt{\left(\frac{\left(a+b+c\right)\left(-a+b+c\right)\left(a-b+c\right)\left(a+b-c\right)}{16}\right).}$$  

The volume of the submaxillary gland using Solidworks^®^ software was estimated as if it were conformed by an ellipsoid body.

Furthermore, using SPSS^®^, the means and standard deviation for each measurement we also calculated.

## Results

### Anatomical measurements of the porcine model

Table 1 lists the anatomical measures, means, and standard deviation for each surgical landmark.

### Head and neck surgery training guide on the porcine model

#### ***Tracheotomy (***Fig. 1***)***

**Fig. 1:**
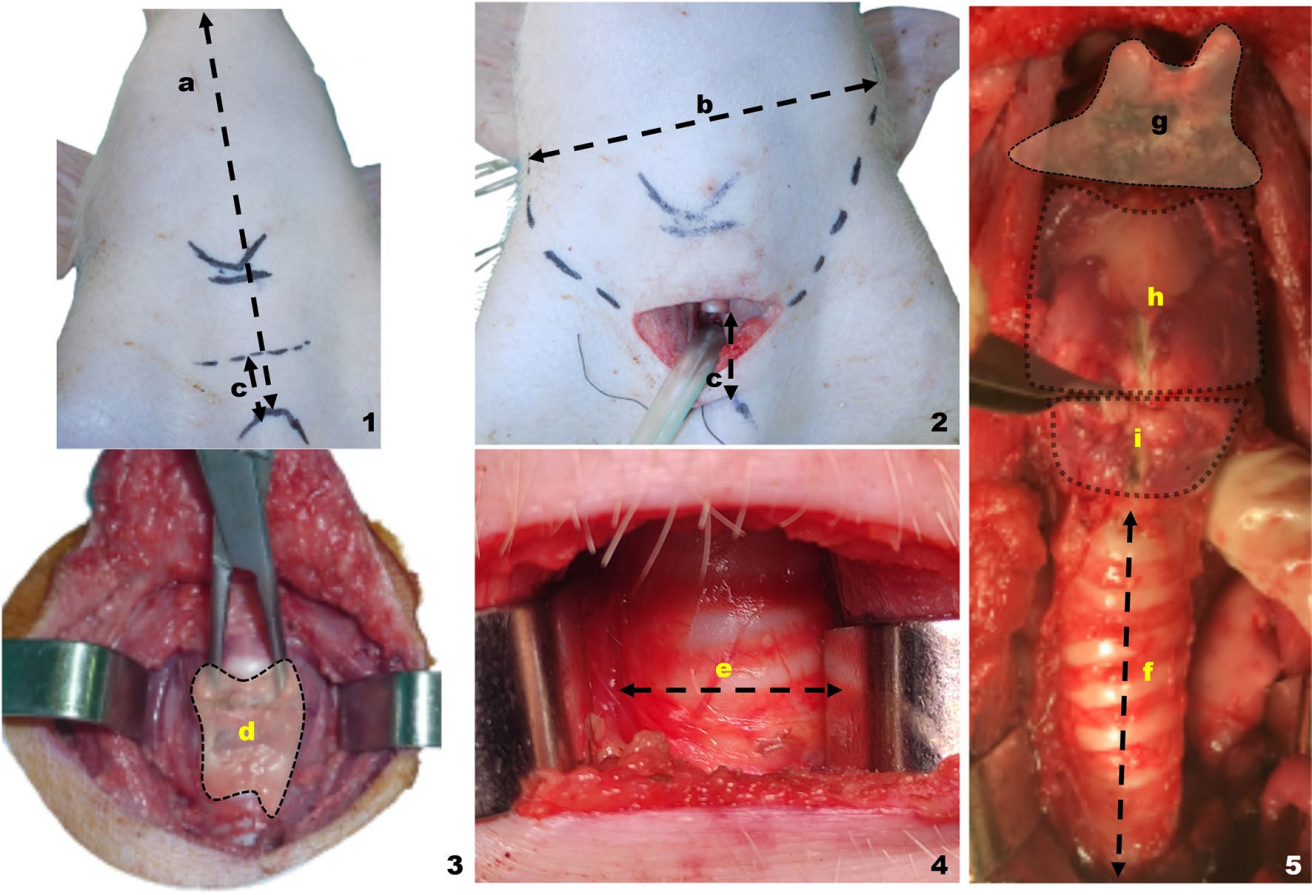
Tracheotomy on the live porcine model. Superior view. **a** Chin–sternum distance, **b** distance between mandibular angles, **c** tracheal incision–sternum distance, **d** thyroid gland detail, e tracheal diameter, f tracheal length, g hyoid detail, h thyroid cartilage detail, i cricoid cartilage detail

All limbs of the porcine model must be fastened and flexed in the supine position, and an appropriate endotracheal tube fixation should be employed (Fig. 11).

Before the training session begins, a number of the animal model peculiarities must be taken into consideration. The sternal-mental distance (*X* = 15.8 cm, *σ* = 0.45) is clearly greater than human and may lead to confusion during the first training steps of the trainee. The mental–mento distance is also greater than in humans (*x* = 11 cm, = 2.3) (Fig. 12).

**Fig. 2:**
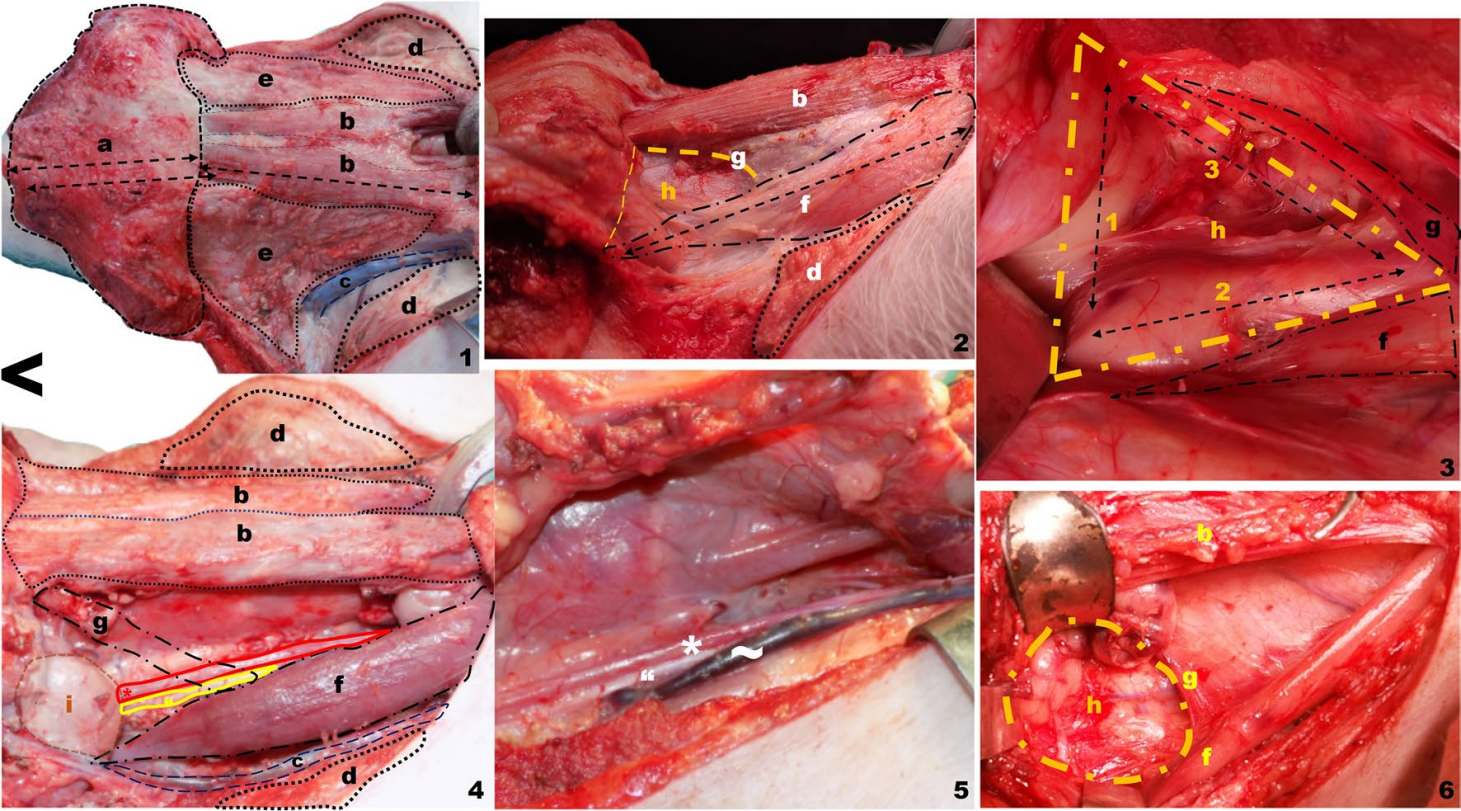
Platysmal flap elevation, neck dissection and spinal triangle exposure on the live porcine model. Right lateral view. < indicates in all the photos the most cranial plane. **a** Platysmal flap length, **b** prelaryngeal (sternohyoid) muscle, **c** external jugular vein, **d** cleidooccipital muscle, **e** brown fat and thymic tissue, **f** sternomastoid muscle, **g** omohyoid (cropped in image 4 to allow the jugular, vagus and common carotid veins to be seen), **h** supraomohyoid triangle: functional neck dissection, **i** submaxillary gland *. Common carotid artery, “. Vagus nerve, ~ . Internal Jugular vein

Tracheotomy has been shown in the swine model to be an excellent procedure for placing residents who are just beginning to develop these capabilities in a practical scenario [6, 9, 10]. The incision for the tracheostomy should be three finger widths above the upper sternal limit.

If the primary goal of the exercise is the tracheotomy, the dissection is remarkably similar to that done on humans at this point. The prelaryngeal muscles are divided and separated in the midline, with the sternohyoid being the dominant muscle (Figs. 14, 2, 1b).

The thyroid gland can be seen when the prelaryngeal musculature has been separated. We have discovered every time during our dissections that the thyroid gland in pigs is noticeably smaller and lower located in the neck than it is in humans. The thyroid gland has a significantly more friable consistency and is situated in the space between the fifth and sixth tracheal rings. This restricts how thyroid surgery could be trained in the porcine model. Additionally, at this level, the most lateral part of the dissection contains an abundance of thymic tissue, which we can be mistaken for thyroid tissue.

Once the thyroid gland has been ligated, a tracheotomy may be performed at this level that reminds to the one performed in humans. Since the tracheal diameter is comparable to that of a human (*x* = 1.3 cm, *σ* = 0.30), standard tracheostomy cannula (Shiley^®^ nº8) or Montandon^®^ tubes are suitable for use (Figs. I2, 31). The most significant difference between this swine model and the human being is that we now find ourselves at a more caudal level, where we can see the tracheal cartilages from the third to the seventh.

**Fig. 3:**
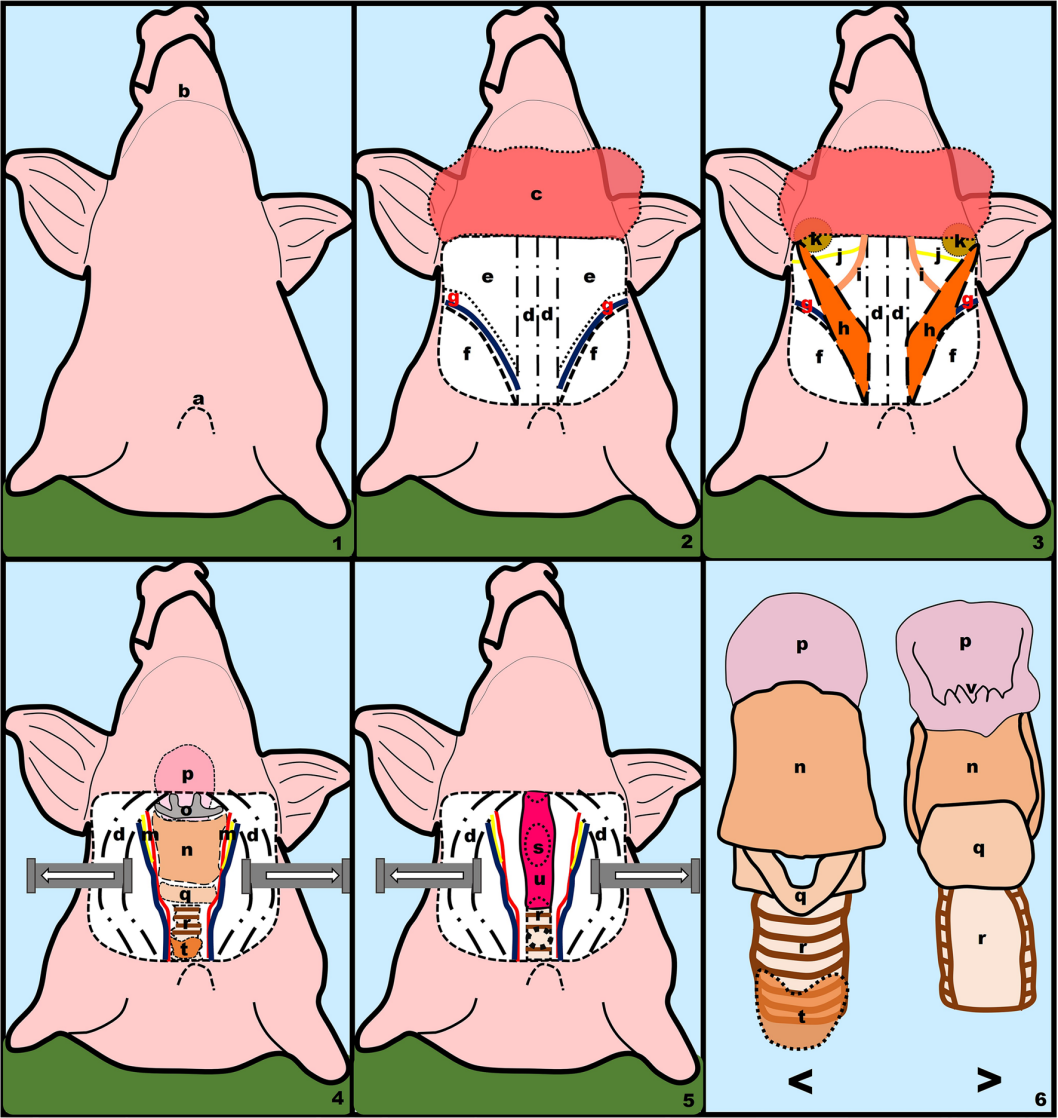
Schematic view of the surgical dissection on the porcine model. Tracheotomy, cervical dissection and total laryngectomy. 1. Porcine model, superior view, 2. platysmal flap elevation, 3. removal of thymic tissue and brown fat, prior to initiating cervical dissection, 4. anterior view of the larynx and trachea after separation of the prelaryngeal musculature, 5. view of the posterior esophageal wall after total laryngectomy, 6. detail of the laryngeal and tracheal structure < : anterior view, > : posterior view. **a** Upper border of the sternum, **b** lower mandibular arch, **c** platysmal flap, **d** prelaryngeal muscles (sternohyoid), **e** brown fat and thymic tissue, **f** cleidooccipital muscle, **g** jugular vein external, **h** sternomastoid muscle, **i** omohyoid muscle, **j** spinal nerve, **k** submaxillary gland, **m** vasculonervous bundle (internal carotid artery, internal jugular vein and vagus nerve), **n** thyroid cartilage, **o** hyoid bone, **p** epiglottis, **q** cricoid cartilage, **r** trachea, **t** thyroid gland, **s** esophageal diverticulum, **u** posterior esophageal wall, **v** vocal rudiment

#### Visceral space and cervical musculature: neck dissection (Figs. 2, 42, 43 and 5)

**Fig. 4:**
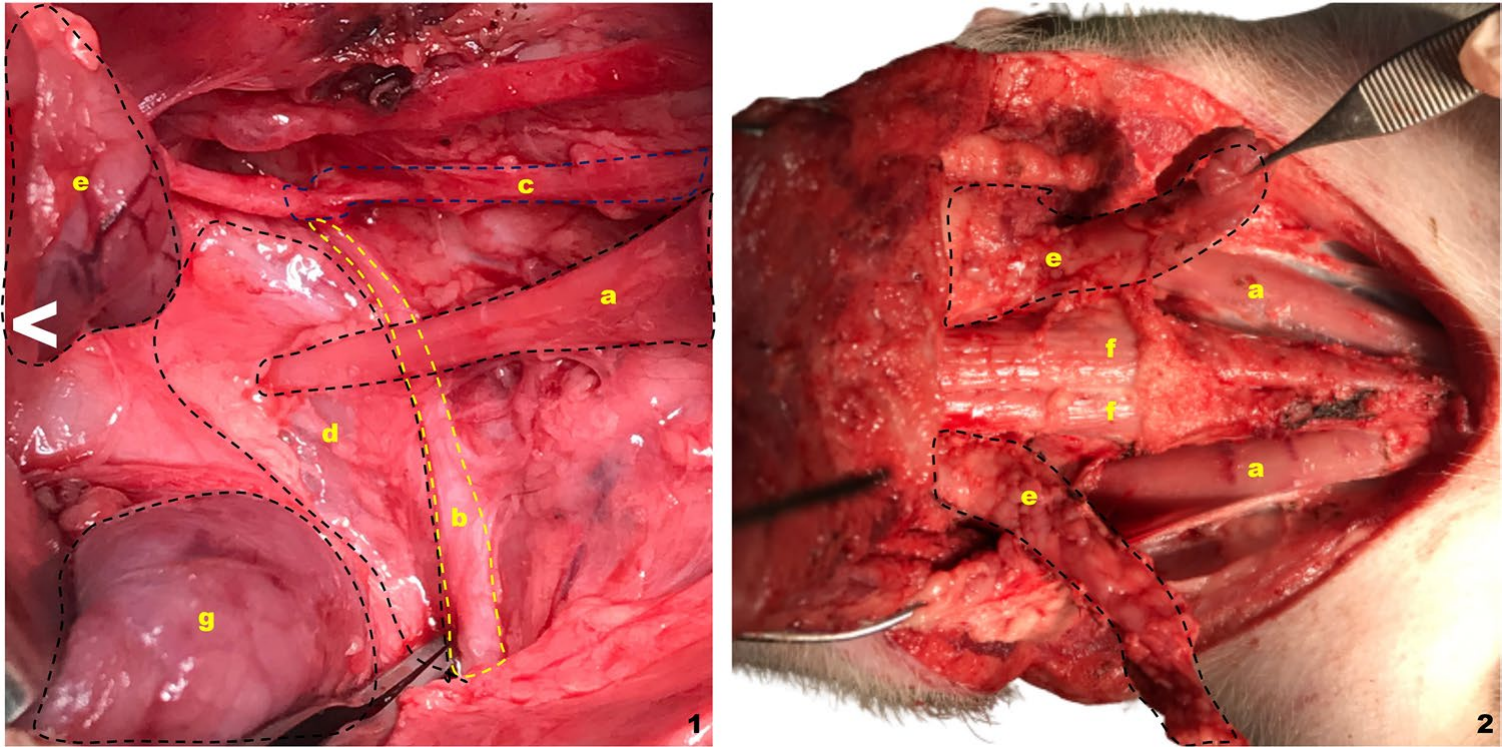
Spinal triangle detailed on the live porcine model Right lateral view (1) and platysmal flap raising (2). < indicates in all the photos the most cranial plane. **a** Sternomastoid muscle, **b** spinal nerve, **c** internal jugular vein, **d** supraspinal space, **e** brown fat and thymic tissue, **f** prelaryngeal musculature, **g** submaxillary gland

**Fig. 5:**
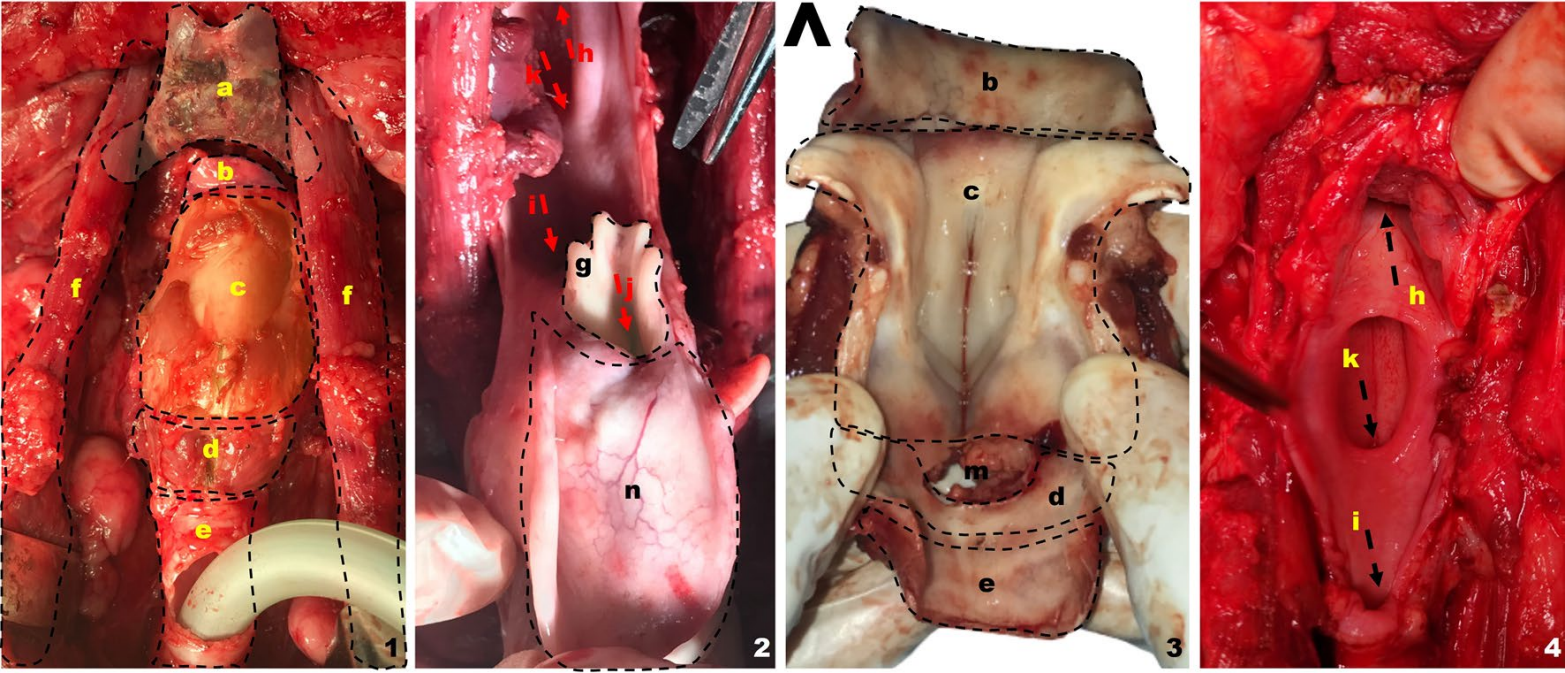
Total laryngectomy on the live porcine model. Superior view. ^ indicates the most cranial end in all images. **a** Hyoid bone, **b** thyrohyoid membrane, **c** thyroid cartilage, **d** cricoid cartilage, **e** trachea, **f** prelaryngeal musculature (separated to the sides), **g** vocal rudiment, **h **direction towards the oropharynx, **i** direction towards the esophagus, **j** glottic space, **k** direction towards the esophageal diverticulum, **m** cricothyroid membrane, **n** epiglottis (everted)

After making the U-shaped incision, the platysmal flap will be raised. The pig model's cervical musculature is strong, thick, and occasionally coated with brown fat in its most superficial area. This fact must be considered when raising the flap, and the brown fat must not be damaged (Fig. 21), otherwise we run the danger of losing the anatomical references. If these prior foundations are ignored, this step may be rather difficult.

Once the platysmal flap is raised we will be able to see the complete extension of the prelaryngeal muscle, the cleidooccipital muscle, as well as all of the brown fat and the thymic tissue mixed in with it. The most lateral component of the platysmal flap raising will include a partial section the cleidooccipital muscle. The cleoidooccipital muscle is located in the most caudal and lateral neck region (Fig. 2, d). The sternomastoid muscle (Fig. 2, f) is located medial to the cleidooccipital muscle and is covered with brown fat. When performing the training session, we shall think about the sternomastoid muscle as if it were the sternocleidomastoid muscle in the human being. The internal jugular vein may be seen crossing between the cleidooccipital muscle and the sternomastoid muscle (Fig. 24).

At this point, there are two possible approaches to the dissection:

- Removing all brown fat and thymic tissue before starting the neck dissection.
- Combining the visceral tissue with the thymus and the brown fat as part of the neck dissection (Fig. 52)

The swine model contains a lot of thymic tissue, which is a significant component of the visceral region of the neck area. In our experience, the later alternative would be confusing for a less trained surgeon, so we advise against using it.

The omohyoid muscle, digastric muscle, and sternomastoid muscle all serve as the margins of the supraomohyoid triangle, which corresponds to functional neck dissection in humans. Because of the extremely small size of this triangle (*x* = 7.07 cm^2^, *σ* = 3.91), the omohyoid muscle can be cut and combined with the visceral dissected tissue (Fig. 24g). The reason the omohyoid muscle is more horizontal in the porcine model and leaves a smaller supraomohyoid triangle is attributed to a significantly more cranial scapula and a shorter neck in a position of sustained flexion.

It is noteworthy how challenging is to identify the digastric muscle during surgical dissection in pigs. This is because the muscle in question has its origins in the jugular process of the occipital bone and inserts itself in a little fibrous bundle in the body of the mandible. Furthermore, in the porcine model, the digastric muscle has only one belly [11]. Therefore, we chose to draw a straight horizontal line from the insertion of the omohyoid muscle to the insertion of the sternomastoid muscle to estimate the area of the supraomohyoid triangle (Fig. 23).

The huge porcine submandibular gland (*x* = 6.16 cm^3^, *σ* = 0.70) needs to be retracted to reveal the spinal nerve crossing under the sternomastoid muscle clearly (Fig. 51b). Given that the spinal nerve's path and placement are identical to those of a human, it is perfect for replicating the surgical steps in this area.

The internal jugular vein and internal carotid artery, along with the vagus nerve, will be seen once the neck dissection is completed. Internal jugular vein (*x* = 0.33 cm, *σ* = 0.049) and common carotid artery (*x* = 0.38, *σ* = 0.13) diameters, in this case, are noticeably smaller than those in humans.

#### Larynx structure (Figs. 3, 44, 46)

The porcine larynx has a tubular shape and is made up of cartilage, perichondrium, connective tissue, and mucus, just like the human larynx. It is divided into the supraglottis, glottis, and subglottis and exhibits the same structural elements as the human glottis: the thyroid cartilage, the cricoid cartilage, and some primitive arytenoid cartilages.

The hyoid bone, which is located above the larynx, has two lesser and two greater horns that are longer than human horns but easily disarticulable (Fig. 31a).

In comparison to humans, the thyroid cartilage is larger. It is shield-shaped, but it lacks the greater horns and the triangular notch (Fig. 31). Furthermore, the thyroid cartilage is connected to the thyrohyoid membrane that is in contact with the hyoid bone (Fig. 31).

The cricoid cartilage is less defined in comparison to humans. Despite this, it has an oval-shaped posterior base ring that articulates with thyroid cartilage in its anterior and superior face via the cricothyroid membrane. The cricoid cartilage continues with the trachea at a lower level and it may be difficult to distinguish its inferior limit from the first tracheal ring.

The epiglottic cartilage is larger than in humans and less rigid. When making an incision through the thyrohyoid membrane to reach the epiglottis, it is common to miss its free border. To evaluate the entire epiglottis, insert a finger into the incision made at the thyrohyoid membrane level and feel for the base of the tongue until reaching this cartilage, which is sometimes flexed and inverted.

We discovered a rudimentary epiglottic cartilage formed by an arytenoid cartilage and some interarytenoid cartilages in the porcine phonatory model, forming what we could call the vocal rudiment (Fig. 3g). The vocal ligament structure is similar to that of humans, but it is much more verticalized.

When the larynx is removed, we can see a diverticulum on the posterior wall of the esophagus (Fig. 34). Although the deficit left by total laryngectomy is slightly greater than in humans, the esophageal stump can be closed.

## Discussion

In recent years, there has been a paradigm shift in the treatment of H&N cancers. Radiotherapy, immunotherapy, and chemotherapy have gradually replaced surgery as the first-line treatment option, particularly in cases where a total laryngectomy was previously required [1, 12–15]. Surgery is now mostly reserved for salvage cases, recurrences, and patients who have already been treated with radiation. The complexity of these procedures, as well as the likelihood of subsequent complications, is unquestionably increased, as are the length of stay, morbidity and mortality, and hospital readmission rates [16, 17].

While hospitals with a high volume of H&N cancer surgery can maintain better surgical standards due to their high specialization, small and medium-sized hospitals, such as those in developing countries, can have a higher rate of complications due to fewer resources [18, 19].

To take the first steps in H&N surgery, it is critical to reconsider instruction and create a simulation model that can provide the resident with an environment as close to realistic operating room conditions as possible.

There are several options when it comes to selecting a model. The rabbit and sheep animal models are among them [20–22], but the porcine model is more accurate in simulating human cervical and laryngeal tissues. Other options include using non-organic simulators [4, 23] or virtual reality technology that is still in development [24, 25]. On the other hand, the live porcine model, as opposed to a human or animal cadaver, allows the learner to experience all of the realistic situations that could arise in the operating room such as bleeding, increasing the difficulty and realism of the surgical training [26].

A variety of pig breeds can be recommended for surgical training. Similar initiatives have been documented using the Yorkshire pig [6] or the Sus Scrofa Domestica [27], but the Large White is easier to manage due to its smaller size and lower cost [8]. Because there are so many different varieties, drawing meaningful parallels between size measurements taken from various pig model breeds and those taken from humans is difficult.

Despite the enormous number of procedures documented in the porcine model, there is little literature that defines the anatomy oriented to perform these procedures. Several authors [28, 29], have provided detailed descriptions of the laryngeal anatomy and histology of the porcine model, which have produced excellent results and demonstrated that the endolaryngeal surgery training method is efficient, reproducible, and affordable [30].

The porcine model has also been proposed for training in laryngeal and airway reconstructive surgery [27, 31, 32] as well as reconstructive microsurgery, with promising results [6, 20, 33] Another application for this framework is the investigation of potential therapeutic alternatives, such as laryngeal transplantation [34, 35]. In our previous work, we used the porcine model to demonstrate through a standardized questionnaire [36] the improvement in surgical proficiency of residents, adding construct validity to the model.

The first barrier to access we discovered in our instance, despite reviewing the specialized literature on swine anatomy, was the lack of a bibliography that in-depth described the cervical spaces, the surgical anatomy, and its approach [37, 38]. Regrettably, because pig animals and humans do not receive the same surgical care, we were unable to find any references that could be used to compare the two. This fact was the foundation for the dissection instructions we provide in this paper, which may be useful to future facilities using the porcine model as a training tool in head and neck surgery.

Propst et al. produced a manual in the form of a book in 2014 [7], outlining in great detail how to handle the porcine model before, during, and after performing airway surgery. We have a knowledge deficit in this area since they only covered airway surgeries (tracheotomy and tracheal repair), leaving out the remainder of the head and neck procedures.

The primary limitation of this work is the lack of validation, as there has never been a comprehensive description of these techniques before, nor is there a literature documenting the anatomy of the porcine model. Face and content validity [39–42] is the most commonly used method for validating surgical simulation models using questionnaires distributed to experts. We believe that comparing the development of surgical skills between residents who received porcine model training and those who did not is a better option. This comparison, however, might be difficult to perform given the scarcity of examples where surgical expertise can be gained, which is the same reason for beginning this type of training.

Despite the limitations mentioned, we think that this guideline contributes to creating and standardizing the porcine model in head and neck surgery training.

## Conclusions

This experience has enabled us to assess the current state of training and teaching for head and neck surgery procedures in our hospital, with the goal of optimizing the learning curve for H&N resident surgeons.

The practice of H&N surgery on the porcine model is described in detail, with a focus on tracheotomy, cervical dissection, and laryngectomy procedures. Given the characteristics described, the pig model appears appropriate for teaching in H&N surgery. The primary flaw in our study is the model's lack of validity. We believe that our simulation model is efficient, approachable, and extremely engaging as a tool for the professional development of the otolaryngology resident. The difficulties that lie ahead will be in validating it.
